# Measuring biomaterials mechanics with atomic force microscopy. 1. Influence of the loading rate and applied force (pyramidal tips)

**DOI:** 10.1002/jemt.23291

**Published:** 2019-05-20

**Authors:** Andreas Weber, Jagoba Iturri, Rafael Benitez, José L. Toca‐Herrera

**Affiliations:** ^1^ Department of Nanobiotechnology Institute for Biophysics, University of Natural Resources and Life Sciences BOKU Vienna Austria; ^2^ Department of Mathematics for Economics and Business Universitat de Valencia Valencia Spain

**Keywords:** applied load–viscoelasticity, atomic force microscopy, cell mechanics, loading rate dependence

## Abstract

Atomic force microscopy (AFM) is today an established tool in imaging and determination of mechanical properties of biomaterials. Due to their complex organization, those materials show intricate properties such as viscoelasticity. Therefore, one has to consider that the loading rate at which the sample is probed will lead to different mechanical response (properties). In this work, we studied the dependence of the mechanical properties of endothelial cells on the loading rate using AFM in force spectroscopy mode. We employed a sharp, four‐sided pyramidal indenter and loading rates ranging from 0.5 to 20 μm/s. In addition, by variation of the load (applied forces from 100 to 10,000 pN), the dependence of the cell properties on indentation depth could be determined. We then showed that the mechanical response of endothelial cells depends nonlinearly on the loading rate and follows a weak power‐law. In addition, regions of different viscous response at varying indentation depth could be determined. Based on the results we obtained, a general route map for AFM users for design of cell mechanics experiments was described.

## 1 INTRODUCTION

Today, atomic force microscopy (AFM; Binnig, Quate, & Gerber, 1986) has turned into a very widely used experimental technique for both imaging and mechanical characterization of biological materials (i.e., cells; Krieg et al., 2019). Due to its fundamental principle of measuring the interaction of matter with matter, and the capability of measuring at ambient conditions (e.g., in liquid, at a given temperature) this technique offers a high diversity of measurement possibilities (Variola, 2015). In addition, by applying various tip/indenter geometries (colloidal, conical, etc.), an *on demand* tip functionalization (SAMs, polyelectrolytes, ligands, etc.; Iturri & Toca‐Herrera, 2017) as well as a broad range of measuring modes (normal indentation, rheological measurements using vibrations, or time‐dependent measurements), many different properties of the biological material under analysis can be determined (Butt, Cappella, & Kappl, 2005; Franz & Puech, 2011; Kumar et al., 2015; Taubenberger, Hutmacher, & Muller, 2014). This includes for example stiffness, adhesive and viscous properties (Benitez & Toca‐Herrera, 2014; Darling, Zauscher, & Guilak, 2006; Gavara, 2017; Rotsch & Radmacher, 2000).

Among those biomaterials, eukaryotic cells represent a good example of complex hierarchical materials in the μm‐range, composed of intertwined arrangements of macromolecules such as proteins, carbohydrates and lipids. These elements act as the building blocks to form different cellular compartments (i.e., nucleus, membrane…) or, alternatively, another type of crucial inner structures such as the cytoskeleton (Alberts et al., 2014). All these supramolecular arrangements and their different dynamics are tightly controlled by the cell, as they can play a key role in the activation of diverse cellular processes such as growth, movement, division, adhesion, and communication (Fletcher & Mullins, 2010). Indeed, the mechanical properties of cells are mostly governed by a joint action of the cytoskeleton (with its three main components actin filaments, microtubules, and the intermediary filaments), the nucleus and the membrane (with the glycocalyx; Ingber, Wang, & Stamenović, 2014).

According to the three dimensional organization of eukaryotic cells, it is rather obvious that depending on both the measurement location (e.g., above the nucleus or the cytoplasmic region of the rim) and the indentation depth applied one might measure different cell properties. An additional aspect to consider when indenting such a complex material is the relative contribution of the constituent parts it is made of. For instance, the above mentioned 3D network of (macro)molecules are reflected in a wide range of relaxation times to measure (from μs‐ms, for for example, lipids in membranes, to seconds for cytoskeletal features, and to minutes for whole cell movement; Melzak, Moreno‐Flores, López, & Toca‐Herrera, 2011). Furthermore, the length scale to be considered might vary accordingly from the nanometer to the micrometer range. All this leads to the fact that cells show, overall, a complex viscoelastic behavior (Lim, Zhou, & Quek, 2006). Then, the response of cells from the perspective of their constituent materials (including elastic, viscous and plastic components simultaneously) and, by extension, the mechanical properties measured might depend on the applied loading rate, loading force, loading time, and directionality (Efremov, Bagrov, Kirpichnikov, & Shaitan, 2015; Nawaz et al., 2012).

In this work, we determine a framework for measuring the mechanical properties of endothelial cells by means of AFM indentation experiments. The differences in cell response to external mechanical stresses were studied for varying loading rates (from 0.5 to 20 μm/s) and maximum loading forces (from 100 pN to 10 nN). The study was restricted to the predefined usage of a single type of cantilever (geometry is a four sided deltoid pyramid). Hence, the dependence of indentation, stiffness, Young's modulus, viscosity, and material history effects have been evaluated as a function of the initial rate/load values applied. Based on the results obtained, a general route map for AFM users can be established for optimally designing cell mechanics experiments.

## 2 MATERIALS AND METHODS

### 2.1 Cell culture and sample preparation

Human Umbilical Vein Endothelial Cells (HUVEC) were grown in T75 flasks using high glucose Dulbecco's Modified Eagle Medium with stable glutamine, supplemented with 10% Fetal Bovine Serum and 1% penicillin/streptomycin. This cell line was chosen because it is a model anchorage‐dependent cell line. Cells were cultured at 37°C with 5% CO_2_ at maximum confluence of 80%. Prior to AFM experiments, cells were trypsinized using 2 mL TrypLE Express, centrifuged and counted. Borosilicate circular cover glasses (diameter: 24 mm, thickness: 0.08–0.12 mm, Menzel Gläser, VWR, Germany) were rinsed with EtOH, N_2_ dried and cleaned with oxygen plasma (GaLa Instrumente GmbH, Austria). The glass slides were then incubated for 24 hr with 4 × 10^4^ cells suspended in DMEM. For measurements, the medium was changed to Leibovitz's L‐15 medium. Media and other compounds above were all provided by Thermo Fisher Scientific (Waltham, MA).

### 2.2 Atomic force microscopy

**Figure 1:**
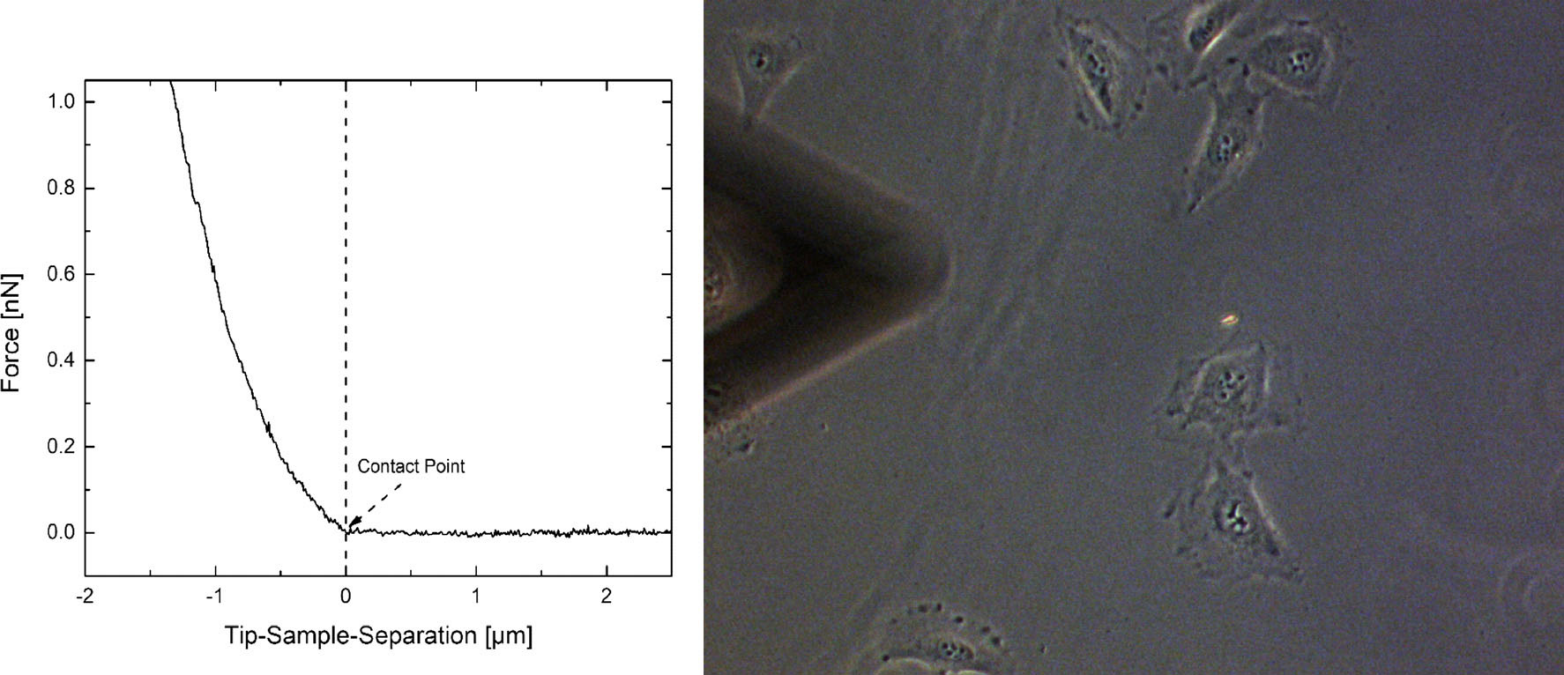
Left. Representative force‐distance‐curve at 5 μm/s with a maximum load of 1 nN. The inset line shows the contact point (see next section for explanation). Right. Optical micrograph (in phase) indicating the indentation position of the cantilever (shown out of focus, black triangle on the left) above the cells by means of red arrows [Color figure can be viewed at wileyonlinelibrary.com]

Measurements were performed on a JPK Nanowizard III (JPK Instruments, Germany) with a CellHesion module mounted on an inverted optical microscope (Axio Observer Z1, Zeiss) at 37°C. Cells were first localized using a ×20 air objective. Triangular, untreated silicon nitride cantilevers with four‐sided pyramidal tips and nominal spring constant of 0.12 N m^−1^ were used (DNP‐S‐B, Bruker). Spring constant calibration was performed using the thermal noise method. For each set of measurements at least 10 cells were measured five times. To test the dependence of cell mechanical properties on loading rate and different fixed applied forces, measurements were performed with a loading rate of 0.5, 2, 5, 10, and 20 μm/s. For each loading rate indentations at following set‐points were performed: 100, 500, 1,000, 2,500, 5,000, and 10,000 pN. Other parameters (curve length, sampling rate) were adjusted according to loading rate and applied load. After 10 indentations, the glass substrate was probed multiple times to ensure tip cleanliness. Before use, cantilevers were cleaned with acetone. Cells were always indented above the nucleus to reduce variability and substrate artifacts. Figure 1 (left) shows a typical F‐d‐curve, taken at 5 μm/s with a maximum load of 1 nN. Figure 1 (right) shows an optical micrograph of the measurement set‐up with the cantilever placed on top of the cells.

### 2.3 Data analysis

Outlier curves were removed after visual inspection. All force curves taken for evaluation can be found in the Supporting Information Figure SI1. The remaining curves were grouped for each loading rate with the respective force set‐point, to evaluate cell‐cell variability (i.e., to test similarity of the curves). The *R afmToolkit* (Benítez, Bolós, & Toca‐Herrera, 2017) was used for data batch‐processing, while Origin Pro9.1 was utilized for data plotting and statistical analysis. The contact point was determined for all curves by optimizing the corresponding parameters in the R afmToolkit, which uses an algorithm described in MRT 2013 (Benítez, Moreno‐Flores, Bolós, & Toca‐Herrera, 2013). This parameter is of crucial importance for evaluation of the Young's Modulus (it is used to calculate the indentation of the sample). The R code for all the calculations performed can be found in the Supporting Information. All numerical data sets were tested for normality (Shapiro Wilk) and for outliers (Grubbs) with significance levels set to 0.05.

### 2.4 Evaluation of the indentation

In AFM force spectroscopy measurements, the overall distance between tip and sample *D* is

(1) $$D=Z_{p}-\left(\delta_{c}+\delta_{s}\right),$$

being *Z*_p_ the position of the piezo, *δ*_s_ the deformation of the sample and *δ*_c_ the deflection of the cantilever. The deflection of the cantilever is proportional to the applied force, *F*, following Hooke's law (note that negative signs are not considered)

(2) $$F=k_{c}\delta_{c},$$

where *k*_c_ is the spring constant of the cantilever and *δ*_c_ is the deflection in the *Z*‐direction. Then, in contact with the sample (*D* = 0) we obtain,

(3) $$\delta_{s}=Z_{p}-\delta_{c}=Z_{p}-\frac{F}{k_{c}}.$$

Therefore, the measured values of piezo position *Z*_p_ and cantilever deflection *δ*_c_ can be used to determine the indentation (deformation) of the sample. The cantilever deflection is measured by the positioning of a laser beam reflected from the cantilever backside to a four‐sided photodiode.

### 2.5 Young's modulus evaluation

A Hertzian contact model with Sneddon extension for the four‐sided pyramidal indenter geometry was used, following

(4) $$F=\frac{E}{1-\upsilon^{2}}\frac{\tan\left(\alpha \right)}{\sqrt{2}}\delta^{2},$$

being *F* the force, *E* the Young's modulus, *v* the Poisson ratio (set to 0.5 for endothelial cells, therefore assuming incompressibility), *α* the pyramidal face angle of the indenter and *δ* the indentation (Hertz, 1882; Sneddon, 1965). The main assumptions for this model are: elasticity (and small strains within its limit), homogeneity, constant contact geometry, the contacted body being infinite, isotropic half‐space, and an indenter having a much higher stiffness than the sample. For small indentations (below 10% of the cell height, in this study an average cell height of 5 μm was used) the former assumptions are accomplished. Thus, from Equation 4 one obtains the following simplification:

(5) $$E=\frac{F}{\delta^{2}}\times C$$

Then, by plotting the force against the square of the indentation the value of the Young's modulus can be evaluated. Note that in this case the dependence is strictly linear, being the Young's modulus proportional to the slope of the straight line. This was performed for all loading rates and force set‐points. All F vs δ^2^ plots with the respective fittings can be found in the Supporting Information Figure SI2.

In detail, using the *R afmToolkit*, the average indentation at a given force value and the average Young's Modulus at given indentation were calculated. For indentations below 200 nm, the Young's Modulus was also determined using a parabolic geometry as a matter of comparison (at small indentations the tip appears more round). Note the change in the exponent for the indentation term and the new parameter *R*_*c*_ (tip‐radius of the indenter) that appear in the original Hertz formula for parabolic indenters.

(6) F=4Rc3E1−υ2δ32

### 2.6 Power law rheology

Probing the cell at different loading rates is used to evaluate cell rheological properties by using a power law (Alcaraz et al., 2003; Fabry et al., 2001). This is normally done by performing sinusoidal oscillations of the cantilever in contact, monitoring the time dependent behavior of the cells over a frequency range of 0.1 to 100 kHz (Rigato, Miyagi, Scheuring, & Rico, 2017). Indentation experiments can be also thought as oscillatory measurements, when one uses not the distance but the time as parameter. Then, the possibility is to determine the relation of an apparent modulus to an indentation rate, which can be described by the following power law (Nawaz et al., 2012)

(7) $$k\left(f\right)=A\times f^{\alpha }$$

with the power law exponent α ranging from around 0.1 to 0.4. The value of the exponent changes according to indentation depth and probing position because of the different viscous properties of the cell constituents. In turn, the indentation rate, *f* (s^−1^), is defined as

(8) $$f=\frac{1}{2\times \left(t_{\delta_{2}}-t_{\delta_{1}}\right)},$$

where *t*_*δ*2_ and *t*_*δ*1_ are the times at which indentations *δ*_1_ and *δ*_2_ are reached.

## 3 RESULTS

### 3.1 Indentations at different loading rates lead to changes in apparent cell stiffness

**Figure 2:**
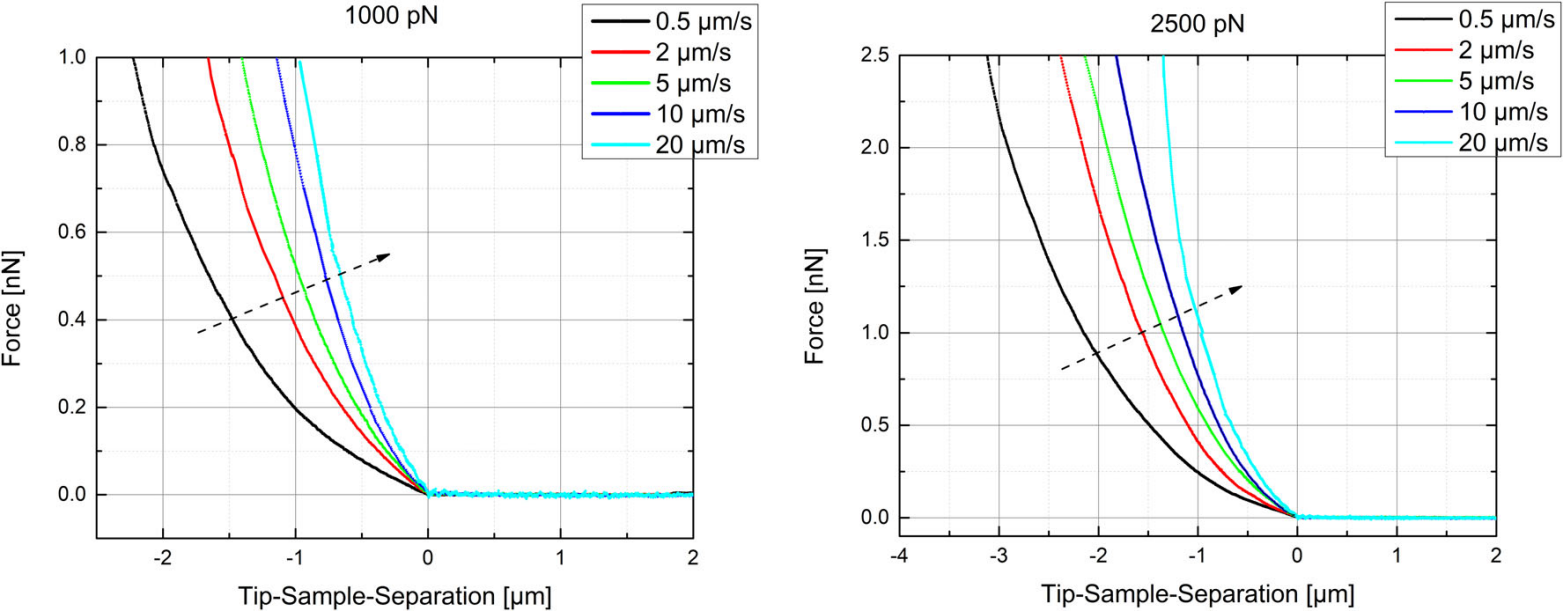
Averaged force distance curves for maximum load of 1 nN (left) and 2.5 nN (right) for varying loading rates. The dashed arrows indicate the increase of stiffness with higher loading rates [Color figure can be viewed at wileyonlinelibrary.com]

The response of HUVEC cells to different loading rates (ranging from 0.5 to 20 μm/s) and varying maximum loads (0.1 to 10 nN) was investigated. The full set of force‐distance curves thus obtained can be found in the Supporting Information Figure SI1. A quick glimpse over these plots brings two immediate visual features that are worth commenting. First, very low loads of 0.1 nN led to bad signal‐to‐noise ratios due to the inherent noise of the AFM system measuring in liquid at 37°C. Second, higher loading speeds led, overall, to higher noise levels due to cantilever vibrations and viscous drag of the medium. In the particular case of a 0.5 μm/s rate, the long measuring times and the noise from the system (floating cells, floating particles, dirt…) provoked the removal of around 50% of the F‐d–curves. Figure 2 shows the averaged *F*‐*δ*–curves obtained for increasing loading rates at two fixed applied forces: 1000 pN (left) and 2,500 pN (right). For both, an increase in stiffness (slope of the curve) is measured for higher loading rates, as explained by the resulting larger slope.

As a consequence, at lower loading rates deeper cell indentations are needed to reach the same applied force. A full comparison of the mean indentation depths obtained for the whole range of loads at different rates can be found in the Supporting Information Figure SI3. Table 1 shows the mean indentation values (±*SEM*, in μm) for each load/rate pair. For the coupled settings of very high forces and the slowest approaching speeds, the resulting indentation was that large (>3.5 μm) that a much stiffer material was sensed by the cantilever. Such an effect probably corresponded to a combined action of the cell nucleus and the underlying stiff glass substrate (in the range of GPa) This can be seen in the Supporting Information Figure SI4, for curves at 0.5 μm/s with a load of 10 nN.

**Table 1:** Mean indentations (in μm, ± *SEM*) of HUVEC cells for different loading rates at different forces

		Cantilever approach rate				
		0.5 μm/s	2 μm/s	5 μm/s	10 μm/s	20 μm/s
Load (nN) (applied force)	0.1	0.59 ± 0.02	0.37 ± 0.02	0.34 ± 0.02	0.26 ± 0.01	0.20 ± 0.02
	0.5	1.65 ± 0.02	1.13 ± 0.03	0.97 ± 0.02	0.77 ± 0.02	0.66 ± 0.03
	1	2.32 ± 0.03	1.63 ± 0.05	1.41 ± 0.02	1.12 ± 0.03	0.98 ± 0.03
	2.5	3.24 ± 0.03	2.40 ± 0.03	2.15 ± 0.07	1.82 ± 0.06	1.35 ± 0.05
	5	3.31 ± 0.06 4.32 ± 0.10	3.23 ± 0.10	2.92 ± 0.11	2.56 ± 0.12	1.97 ± 0.06
	10	n.a.	n.a.	3.35 ± 0.17	3.17 ± 0.04	2.61 ± 0.08

Interestingly, indentation experiments at a constant loading rate while varying the applied forces did not affect the load history of the sample. Even at a force of up to 10 nN (leading to indentations above 50% of the cell height) with the sharp, pyramidal indenter, cells are able to recover their prior mechanical stability. Thus, the first *F*‐*d*–curves delivered the same indentation values as the subsequent ones at the corresponding force value (note that this was not the same for the opposite situation, when the applied force was constant and the loading rate changed). The increase in apparent stiffness with higher loading rates underlines the fact that cells act as viscoelastic material rather than pure elastic one. As aforementioned, such a viscoelastic response is determined by the different cell components simultaneously contributing with their respective elastic, plastic, and viscoelastic properties. Apparently, the elastic response increases when probing the sample with higher frequencies.

### 3.2 Multiple stiffness regions appear in *F*‐*δ* ^2^ plots

**Figure 3:**
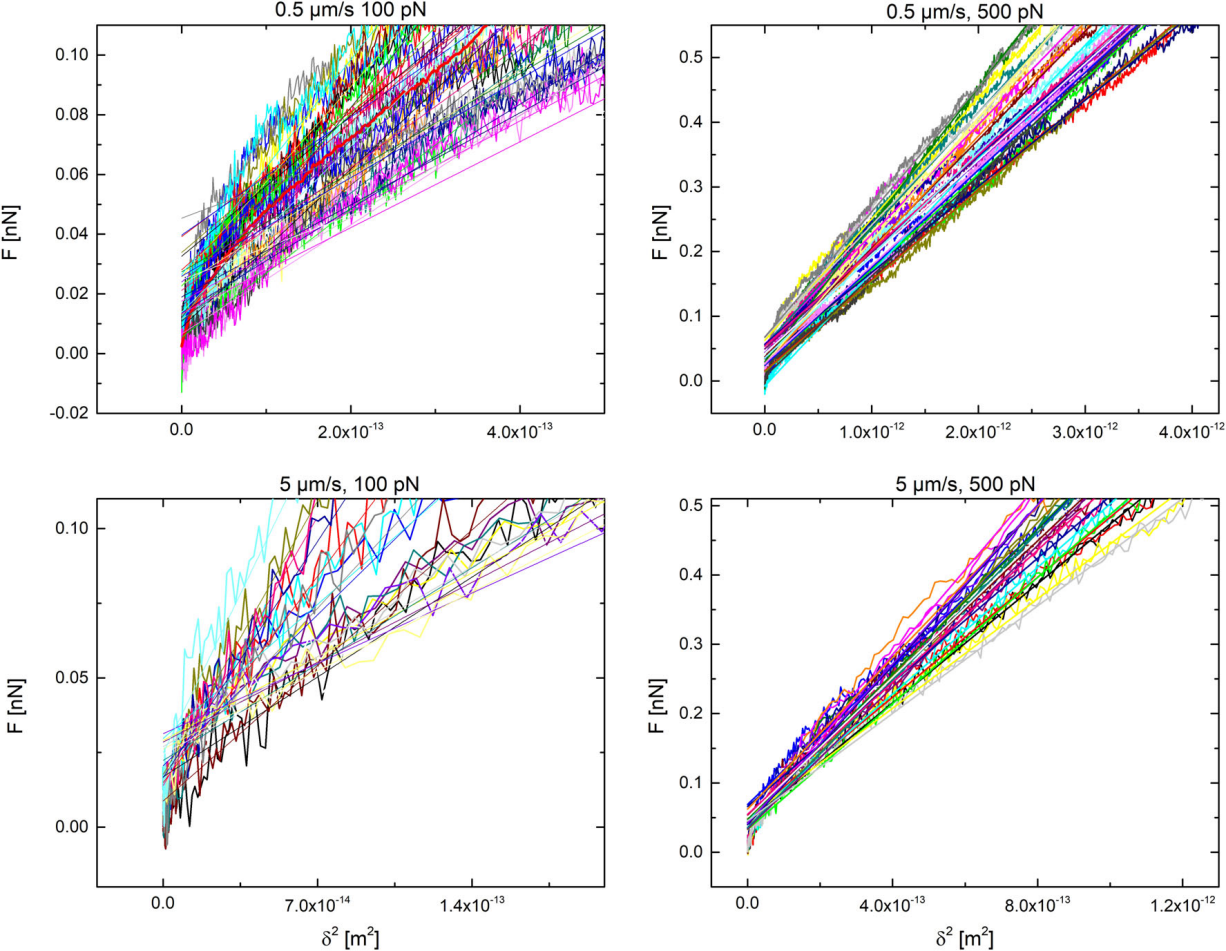
*F* vs. *δ* ^2^ curves including the corresponding linear fittings for 0.5 μm/s at 100 pN (top, left) and at 500 pN (top, right) and for 5 μm/s at 100 pN (bottom, left), and 500 pN (bottom, right). The mean adjusted *R* ^2^ are the following: 0.5 μm/s, 100 pN *R* ^2^ = 0.971; .5 μm/s, 500 pN, *R* ^2^ = 0.991; 5 μm/s, 100 pN, *R* ^2^ = 0.808, 5 μm/s, 500 pN *R* ^2^ = 0.997 [Color figure can be viewed at wileyonlinelibrary.com]

As already discussed in the methods section, the determination of the Young's Modulus when using a pyramidal indenter can be rather easily done by plotting the force versus the squared indentation (Equation 5), where the resulting slope corresponds to that factor. For a parabolic tip geometry one should plot *F* versus *δ* ^3/2^ instead. These plots are also useful to visually detect regions of changing stiffness, based on the slope variation observed. The whole set of *F*‐*δ* ^2^ plots with linear fittings can be found in the Supporting Information Fig. SI2. From these plots, a significant change in the slope was observed for loading rates lower than 5 μm/s and indenting loads above 2.5 nN (see an example in the Supporting Information , Figure SI5). Figure 3 shows the representative *F*‐*δ* ^2^ plots obtained for 0.5 and 5 μm/s at a maximum load of 0.1 and 0.5 nN with the respective linear fitting over the whole data range. A region of different stiffness is shown to appear under both approaching rates for loading forces below 50 pN. For 0.5 μm/s, these forces produced an indentation of around 250 nm, while for 5 μm/s the value was around 200 nm. Therefore, at such low forces (<50 pN) the cell stiffness might be underestimated by merely using the slope‐based approach of the *F*‐*δ* ^2^ curves (a more thorough evaluation of the change in slope can be found in the Supporting Information, Figure SI6). On the contrary, for values above 100 pN the model fitting seemed to be more accurate. Table 2 shows the average slope values calculated for the different loading rate and forces.

**Table 2:** Averaged slopes of *F*‐*δ* ^2^ curves depending on load and loading rate. The relative error is in the range from 3 to 10%

		Cantilever approach rate				
		0.5 μm/s	2 μm/s	5 μm/s	10 μm/s	20 μm/s
Load (nN) (applied force)	0.1	Two linear regimes	417.9	723.8	1,258.4	Not fittable
	0.5	162.2	351.4	494.6	801.9	1,045
	1	181.3	352.2	469.3	828.2	942.9
	2.5	232.4	426.8	560.6	820.2	1,001.9
	5	Not linear	Not linear	Above 3.5 nN not linear	710.14	908.5
	10	Not linear	Not linear	Above 3.5 nN not linear	928.24	1,250.9

From the values in Table 2 a rising trend in the slope values can be seen to occur when the loading rate is increased at fixed load. When this is observed from the opposite perspective (fixed rate and changing load) a decrease of the slope appears between 100 and 500 pN and above 2.5 nN, indicating a reduction of cell stiffness, while the respective values recorded in the intermediate range (0.5–2.5 nN) laid in the error range of each other. The higher stiffness recorded in the extreme cases is thought to derive from two different factors: first, the change in contact geometry (apparent spherical instead of the true pyramidal shape) at very shallow indentations. Second, the existence of the so‐called actin cortex, constituted mostly of actin and connected to the cell membrane with a thickness of around 100 to 250 nm. Being composed of stiff, fibrillary structures, the cortex has a higher stiffness than the underlying cytoplasm and a more viscous appearance. Much care has to be taken at experiments under highest forces, because of possible underlying substrate effects at indentations above 10% of the cell height.

**Figure 4:**
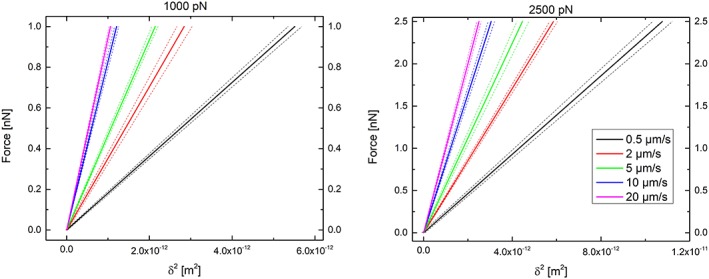
Simulated curves for determined slopes of *F*‐*δ* ^2^ curves for different loading rates at maximum forces of 1 nN (left) and 2.5 nN (right). Note that the largest value of the Young's modulus (slope) is obtained at 20 μm/s (magenta) while the lowest one is obtained at 0.5 μm/s (black) [Color figure can be viewed at wileyonlinelibrary.com]

The slope values calculated in the indenting load region between 0.5 and 2.5 nN (which cover the entire approaching rate range) were used for simulating the corresponding *F*‐*δ* ^2^‐curves, as shown in Figure 4.

### 3.3 Rheological properties depend on indentation depth

**Figure 5:**
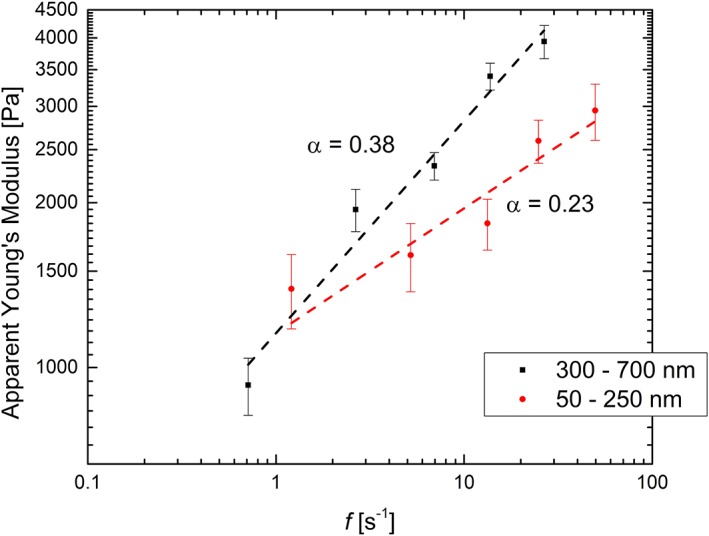
Apparent Young's modulus as a function of indentation rate, evaluated for two different indentation ranges (50 to 250 nm and 300 to 700 nm). The fitting follows the power law as seen in Equation (7), with adjusted *R* ^2^ of .966 for 300 to 700 nm and *R* ^2^ of .888 for 50 to 250 nm. The calculated fitting values are *α* = 0.38 (*A* = 1,156) and *α* = 0.23 (*A* = 1,155) respectively. Additionally, using a pyramidal indenter geometry (not shown above) for the shallow indentations, fitting values of *α* = 0.25 (*A* = 3,345) were determined [Color figure can be viewed at wileyonlinelibrary.com]

Further mechanical analysis was devoted to test the weak power‐law behavior of HUVEC cells at different loading rates (see Section 2). In this experiment, we compared the behavior at both shallow (50 to 250 nm) and slightly deeper (300 to 700 nm) indentations. This led to indentation rates (*f*, in s^−1^, as defined in Equation 8) ranging from 0.5 to 27 s^−1^ for the large indentation and 1.2 to 50 s^−1^ for the shallow one, respectively. For the deeper indentation the cell Young's Modulus could be calculated according to Equation (4), while for the smaller indentation we used both Equation (4) and (6) with a radius of 40 nm (maximum tip radius, provided by the manufacturer). Figure 5 shows the apparent Young's Modulus as a function of the indentation rate, plotted as double‐log.

By fitting the datasets with the power law defined in Equation (7), an exponent of 0.38 is found for the case of a higher indentation, and a reduced exponent of 0.23 for lower ones, both values being in the range of data published in the literature (Hoffman & Crocker, 2009). The change in the power law exponent is related to the viscous nature of the cell with respect to the indentation depth achieved. This behavior might be due to the presence of the actin cortex right beneath the cell membrane, which has a thickness of about to 200 nm. Due to its nature, this cortex is thought to be very viscous (Gardel, Valentine, Crocker, Bausch, & Weitz, 2003). In the case of a deeper indentation, the dependence of apparent Young's modulus on the indentation rate showed a reduction of the viscosity. Thus, it is possible to monitor changes in viscosity of different parts of the cell just by performing “standard” indentation experiments (at different rates).

## 4 DISCUSSION AND CONCLUSIONS

The determination of the mechanical properties of cells depends not only on the technique used, but also on the choice of predefined experimental settings. The aim of this work was to find and establish an experimental framework suitable for determining the mechanical properties of endothelial cells using the AFM and sharp pyramidal indenters. The varied parameters were the loading rate (from 0.5 to 20 μm/s) and the maximum applied load per measurement (from 0.1 to 10 nN). With these experiments we wanted to test the following hypotheses

1. Measured mechanical properties *depend on the loading rate*, showing complex material properties of cells (with elastic, viscous, and plastic components), following a weak power law
2. Different cell properties can be measured at *different indentation depths*
3. Cells can withstand high forces, even when using sharp tips
4. A wide range of rates and loads (applied forces) can be used to test different properties

To give a particular answer to each of the questions above, application of suitable experimental conditions was required (for HUVEC cells), as summarized in Table 3.

**Table 3:** Summary of the applied experimental conditions and the respective outcomes

Conditions	Results
Fixed forces, changing loading rates	Properties change with loading rate (viscoelasticity) Higher loading rates, higher noise levels Low loading rates, long experimental time
Fixed rates, changing forces	Different indentations depths, different cell submaterials are felt (heterogeneity) Too high forces led to nucleus and substrate indentation Too low forces lead to bad signal to noise ratio (with AFM noise around 10 pN) No material history effects (high force also no effects)
Fixed force, fixed speed	For all experiments comparable curves where achieved
Independent of speed and force	Two slopes in *F*‐*δ* ^2^ curves (below 50 pN first, above second) (contact geometry change, actin cortex)
Speed below 2 μm/s, force above 3.5 nN	Substrate (+ nucleus) visible in curves
Indentation rate between different points	Viscosity of material Dependence of viscosity on indentation depth (anisotropy and heterogeneity of cells)

In this work, we have shown the importance of a priori defining measurement parameters for determination of mechanical properties of cells using the AFM as an indentation device. Users should test different loading rates and maximum force values to ensure optimal experimental conditions. From our experience, loading rates ranging from 1 to 10 μm/s work well for endothelial cells, while maximum loads from 250 to 2,500 pN seem to be feasible. Of course the ranges depend on the experiment performed and the cells used. In addition, experimenters should always consider the constraints of the used model to calculate properties and that overall cells are complex, multilayered viscoelastic materials. For example, the main assumptions of the Hertzian contact mechanics model (homogeneity, isotropy, constant contact geometry, sample being an infinite half‐space), are in fact not respected by the cell nature. Nevertheless, this model can be used under concrete assumptions. In addition, extensions of the model or measuring directly viscoelastic properties (such as the thin layer extension or the correction for the underlying substrate [Eric M. Darling, Zauscher, Block, & Guilak, 2007; Gavara & Chadwick, 2012]) can help to make the data evaluation more feasible.

Another point to think about is the time needed for experiments—while some samples are easily reproduced and can be measured many times, there are, of course, also samples with short life‐time. By optimizing the quality of data acquisition of the imaging of the mechanical properties (like force mapping, JPKs QI‐mode, Brukers peak‐force QNM‐mode, …), one should take into account the applied high loading rates (often above 20 μm/s) used for these experiments to ensure a high number of pixels, which will lead to higher apparent stiffness values.

An issue that was not considered in this study was comparison between different cantilevers/probes. Here, the experimenter has a myriad of possible choices. The cantilever stiffness should be in the range of the sample measured (for cell mechanics, normally cantilever stiffness with values ranging from 0.3 to 0.01 N/m are used), while the resonance frequency should be as high as possible. With respect to geometry, the most important choice is to either use a tip, like in this study or to use a spherical particle. The tip can of course be better used in (mechanical property) imaging and has defined indentation localization, while the spherical particles are more widely used for full cell mechanical studies. Here a point to consider is the local pressure put onto the cell, which is much higher when using tips (consider the contact area and the load). This will be the topic of another study.

## CONFLICT OF INTEREST

The authors declare no conflict of interest.

## Supporting information


**Appendix S1**: SUPPLEMENTARY INFORMATION
