# Supplementary material for: Measuring biomaterials mechanics with atomic force microscopy. 1. Influence of the loading rate and applied force (pyramidal tips)
Source: Microsc Res Tech. 2019 May 20;82(9):1392–400. doi: 10.1002/jemt.23291 (PMC6767567; doi:10.1002/jemt.23291)

SUPPLEMENTARY INFORMATION

Title: Measuring biomaterials mechanics with atomic force microscopy. 1. Influence of the loading rate and applied force (pyramidal tips).

Andreas Weber, Jagoba Iturri, Rafael Benitez and José L. Toca-Herrera

SI.1-1 – 0.5 µm/s Force-Distance curves. Force-Distance curves taken with a loading rate of **0.5 µm/s**, for applied forces (loads) of 100, 500, 1000, 2500 and 5000 pN. Note the appearance of another slope when measuring up to 5 nN. The slope change appeared at a force of around 2.5 nN and an indentation of around 3 µm for 30% of the measurements.

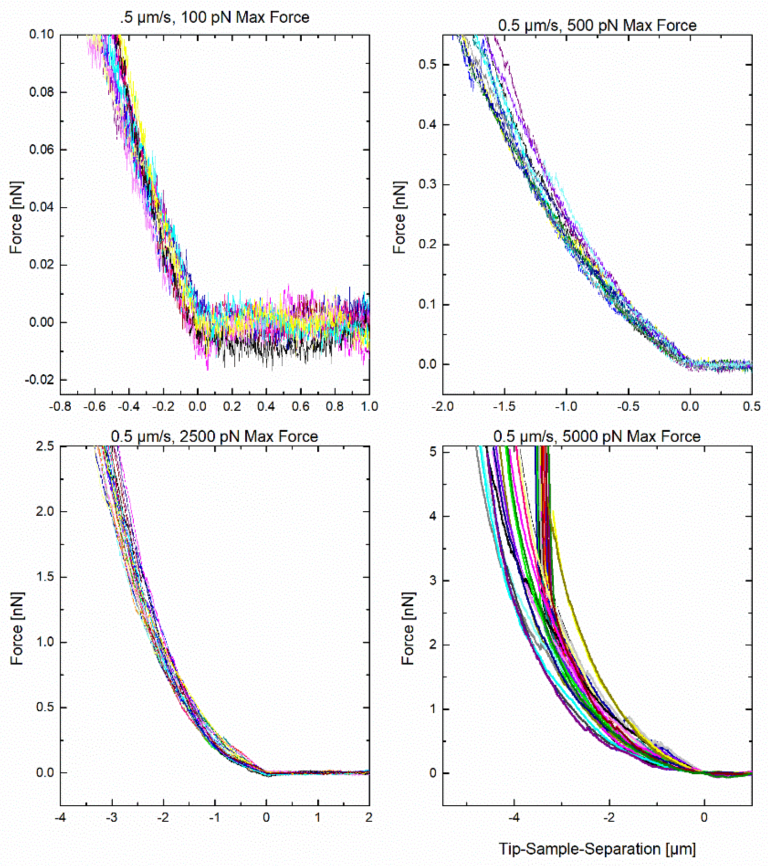

SI.1-2 – 2 µm/s Force-Distance curves. Force-Distance curves taken with a loading rate of 2 µm/s, for applied forces (loads) of 100, 500, 1000, 2500 and 5000 pN. Note the appearance of another slope when measuring up to 5 nN for 10% of the curves. The slope change appeared at a force of around 4 nN and an indentation of around 3 µm.

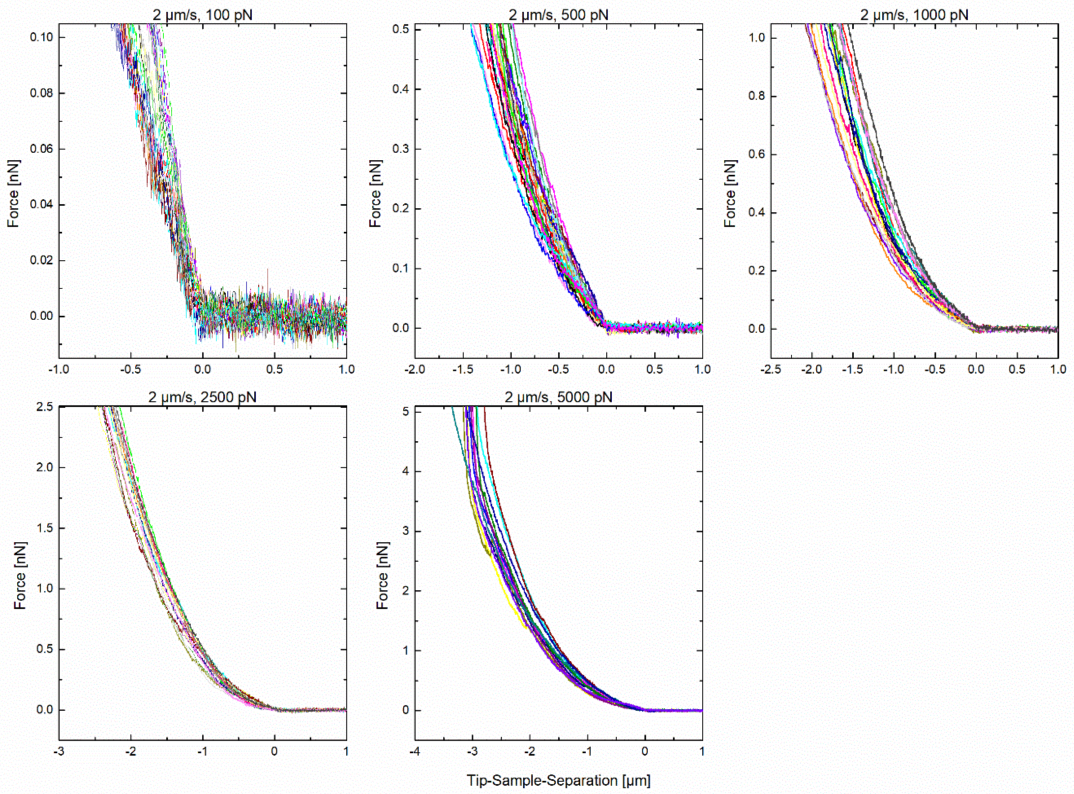

SI.1-3 – 5 μm/s Force-Distance curves. Force-Distance curves taken with a loading rate of **5 μm/s**, for the loads of 100, 500, 1000, 2500, 5000 and 10000 pN. An increase in the noise level was visible. At indentations up to 10 nN, indenting up to 8 nN led to the appearance of another slope (at around 3.5 μm).

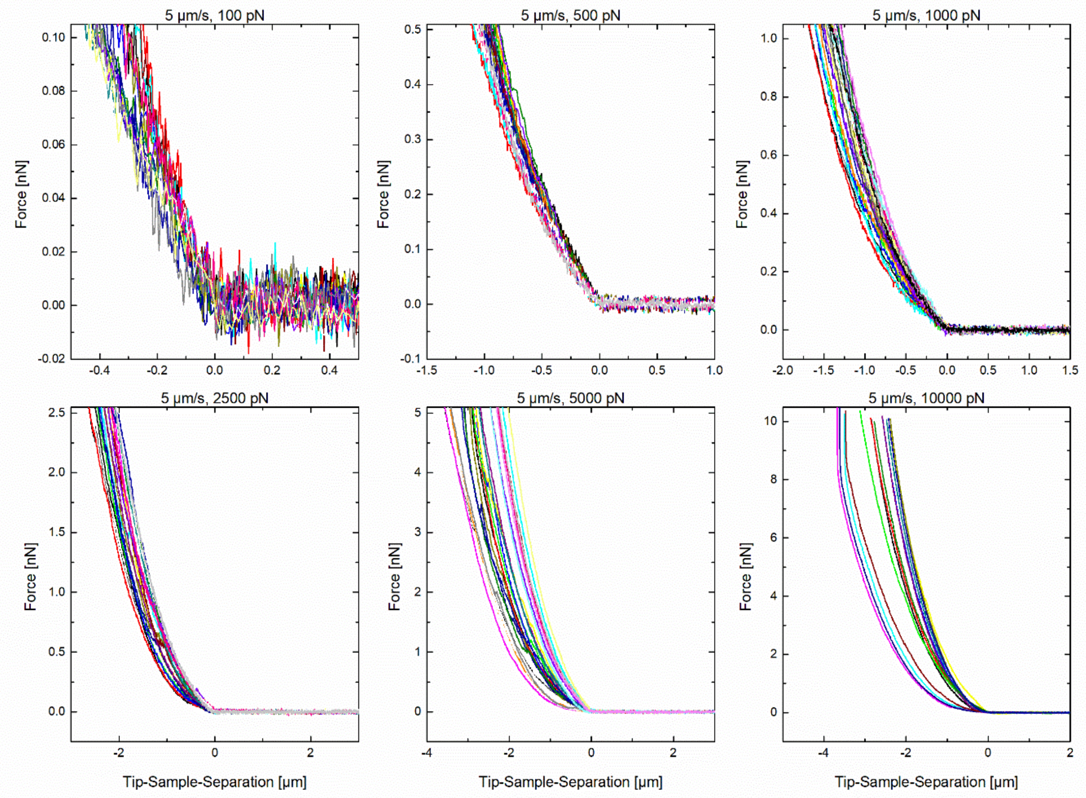

SI.1-4 – 10 µm/s Force-Distance curves. Force-Distance curves taken with a loading rate of **10 µm/s**, for the loads of 100, 500, 1000, 2500, 5000 and 10000 pN. An increase in the noise level was visible. No change in the slope appeared.

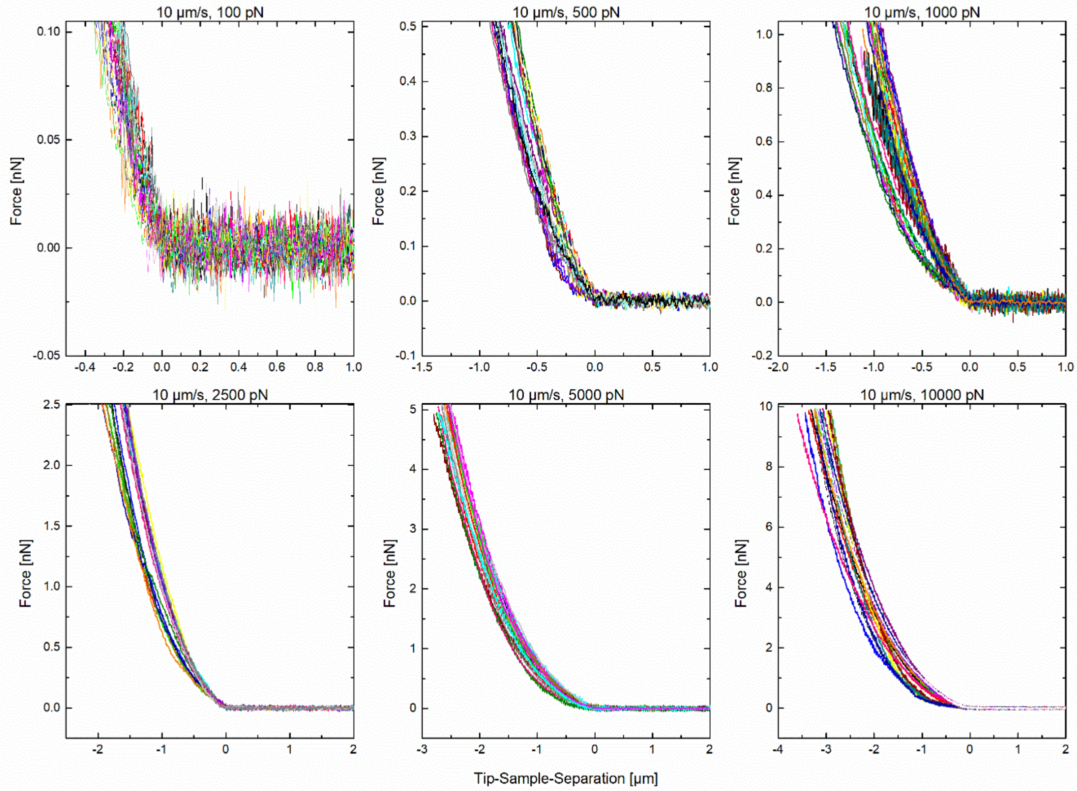

SI.1-5 – 20 µm/s Force-Distance curves. Force-Distance curves taken with a loading rate of **20 µm/s**, for the loads of 100, 500, 1000, 2500, 5000 and 10000 pN. An increase in the noise level was visible. At low forces (100 pN) the signal to noise ratios made fitting nearly impossible. No change in the slope appeared. A higher variability appeared.

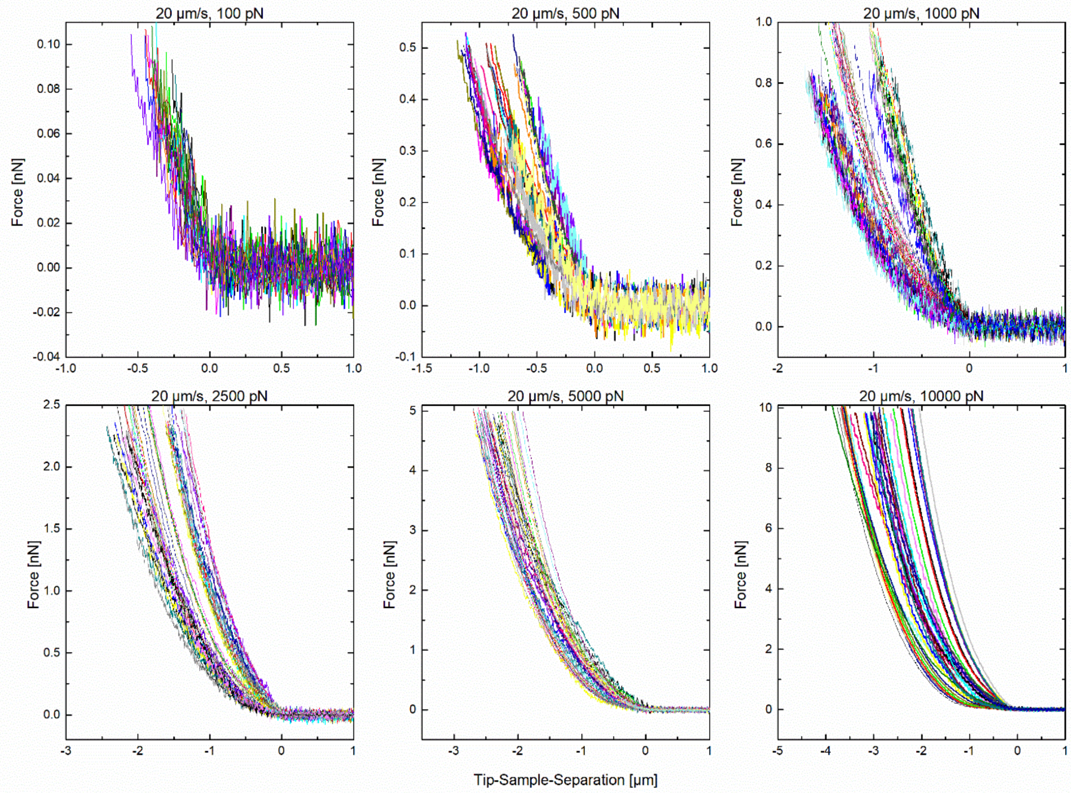

SI.2-1 – F- δ² curves for 0.5 µm/s, 100 to 2500 pN. The figure shows the F-(indentation)²-curves with the respective linear fittings. At forces below 50 pN, another (steeper) slope was visible. Above 100 pN the linear fittings worked very well. Above 3500 pN, another slope appeared (not shown here). Fitting performance (R^2): 100 pN (0.971), 500 pN (0.991), 1000 pN (0.996), 2500 pN (0.992).

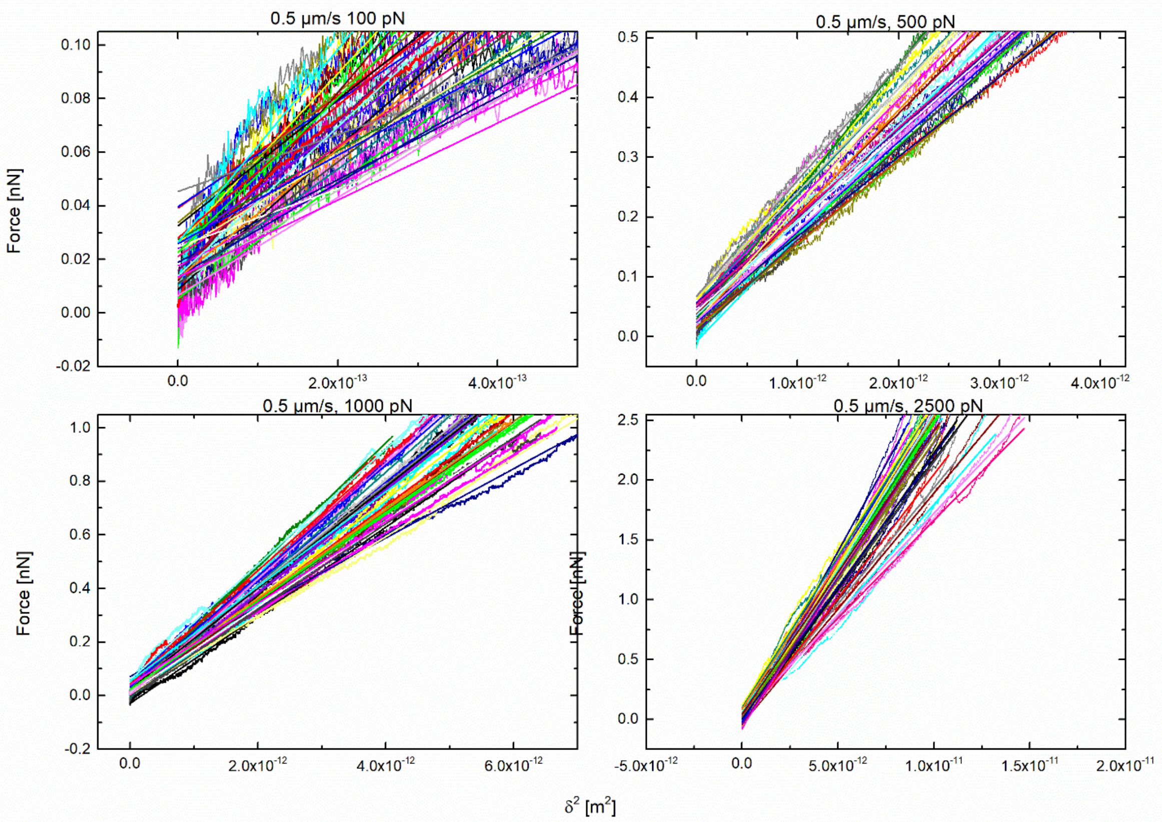

SI.2-2 – F- δ² curves for 2 µm/s, 100 to 2500 pN. The figure shows the force-(indentation)² curves with the respective linear fittings. At forces below 50 pN, another (steeper) slope was visible. Above 100 pN the linear fittings worked very well. Fitting performance (R^2): 100 pN (0.951), 500 pN (0.993), 1000 pN (0.994), 2500 pN (0.995).

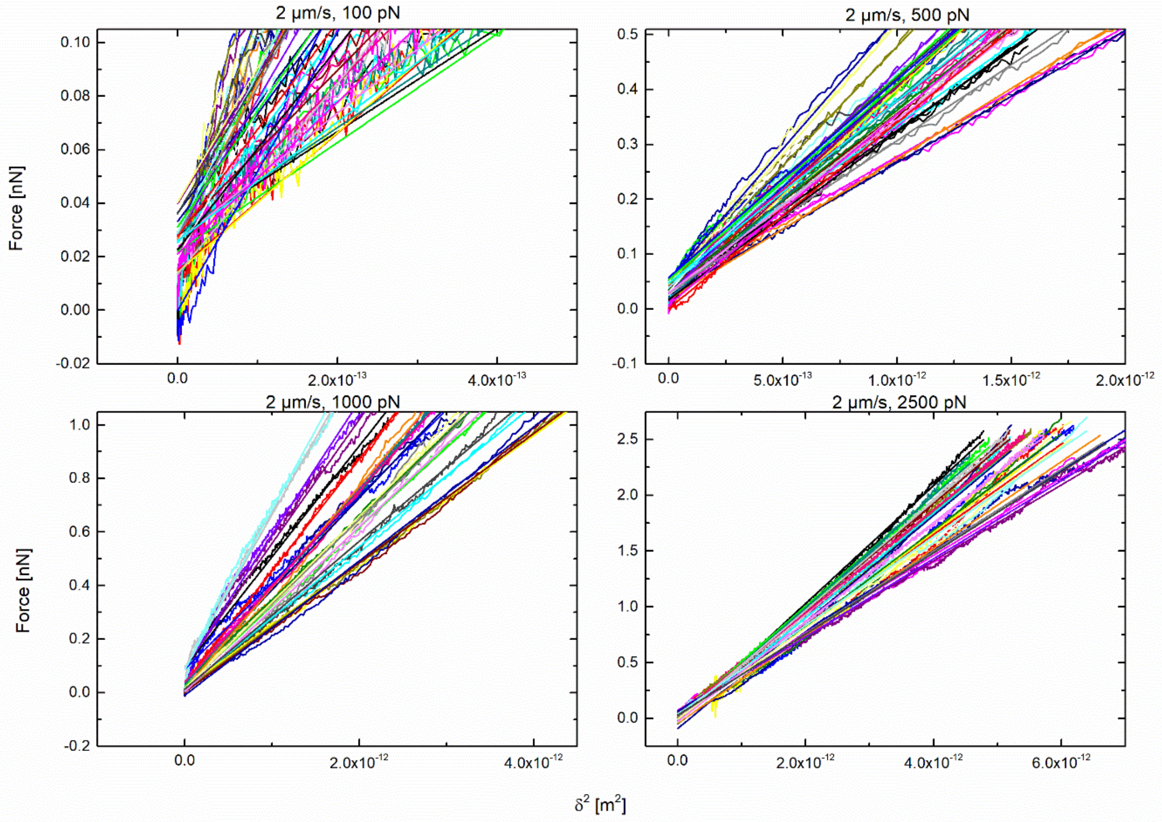

SI.2-3 – F- δ² curves for 5 µm/s, 100 to 2500 pN. The figure shows force-(indentation)² curves with the respective linear fittings. At forces below 50 pN, another (steeper) slope was visible. Above 100 pN the linear fittings worked very well. Note the higher noise levels at this speed. Above 3.5 nN, another slope appeared. Fitting performance (R^2): 100 pN (0.808), 500 pN (0.989), 1000 pN (0.996), 2500 pN (0.995).

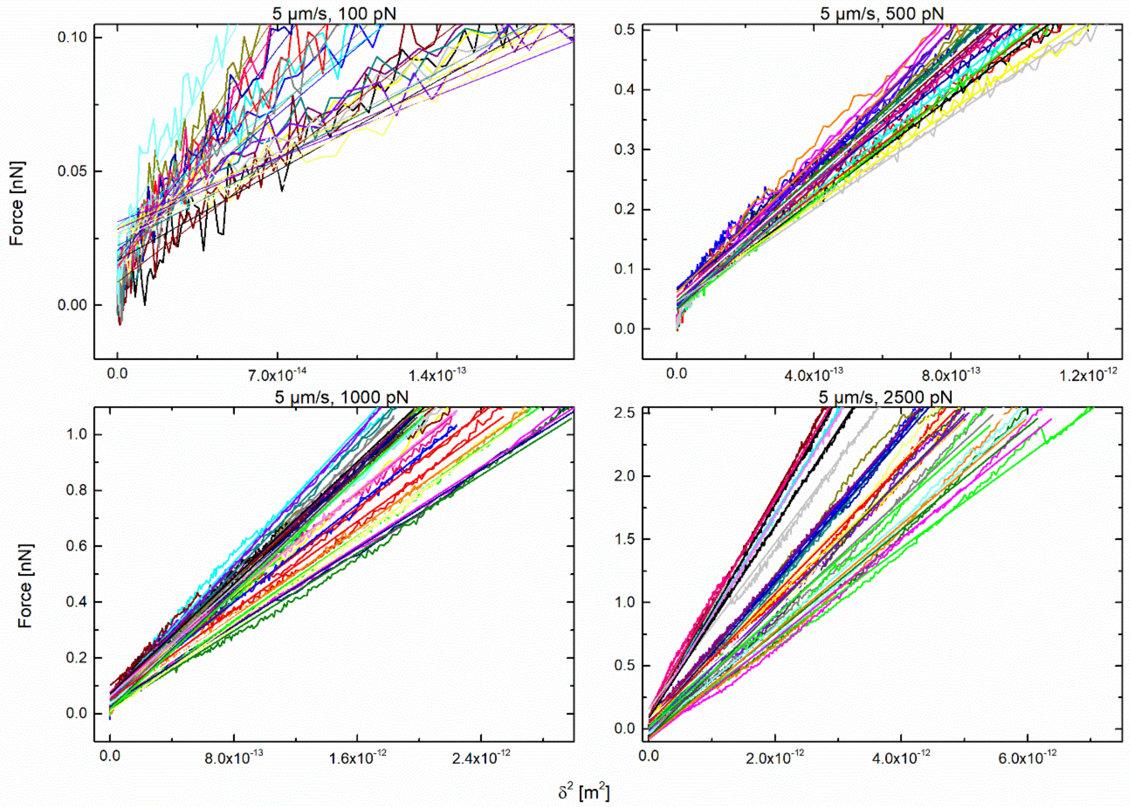

SI.2-4 – F- δ² curves for 10 µm/s, 100 to 10000 pN. The figure shows force-(indentation)² curves with the respective linear fittings. At forces below 50 pN, another (steeper) slope was visible. Above 100 pN the linear fittings worked very well. Note the higher noise levels at this speed. Above 7 nN, another slope appeared. Fitting performance (R^2): 100 pN (0.941), 500 pN (0.986), 1000 pN (0.992), 2500 pN (0.998), 5000 pN (0.995), 10000 pN (0.988).

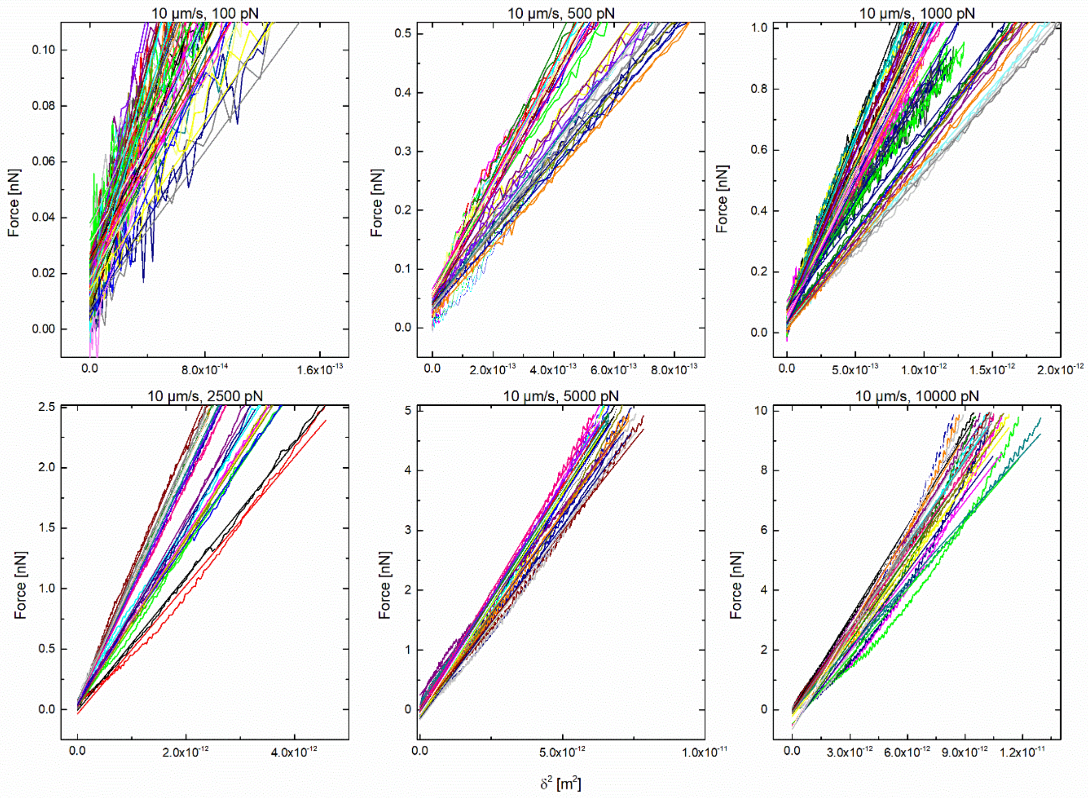

SI.2-5 – F- δ² curves for 20 µm/s, 500 to 10000 pN. The figure shows force-(indentation)² curves with the respective linear fittings. Below 500 pN, curves were not fittable (too high noise). Note the higher noise levels at this speed. For around 10% of the curves at 10 nN max load a different behavior appeared. Fitting performance (R^2): 500 pN (0.971), 1000 pN (0.990), 2500 pN (0.978), 5000 pN (0.994), 10000 pN (0.986).

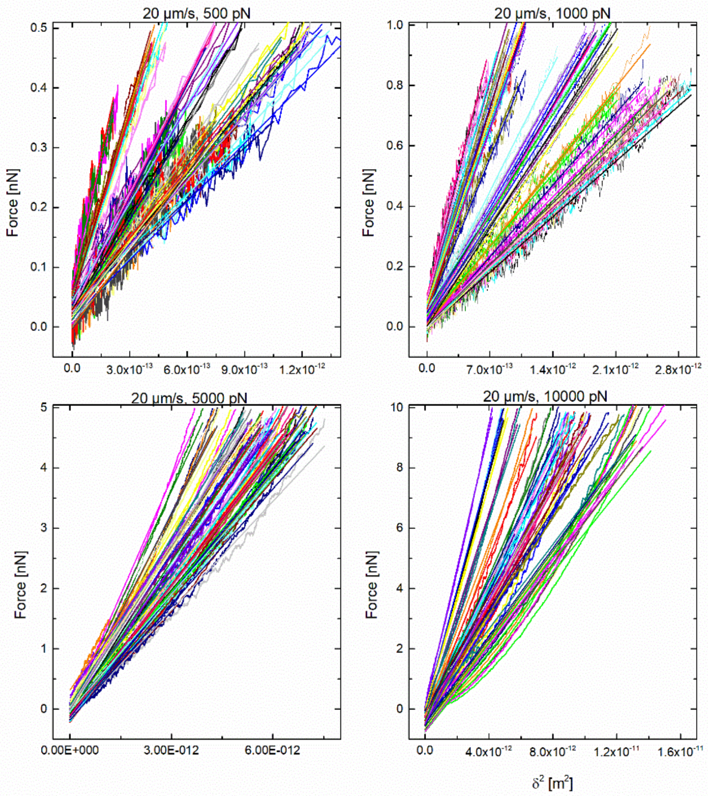

SI.3 – Mean indentation depth for all experimental speeds (loading rates) and applied forces. One experiment consisted of measuring at a given loading rate varying the forces from low to high. Then the next loading rate was tested. Note that for the respective loading rate, similar indentations at the same load were determined. This shows that apparently, no material history is visible, even after indentations of up to 10 nN.

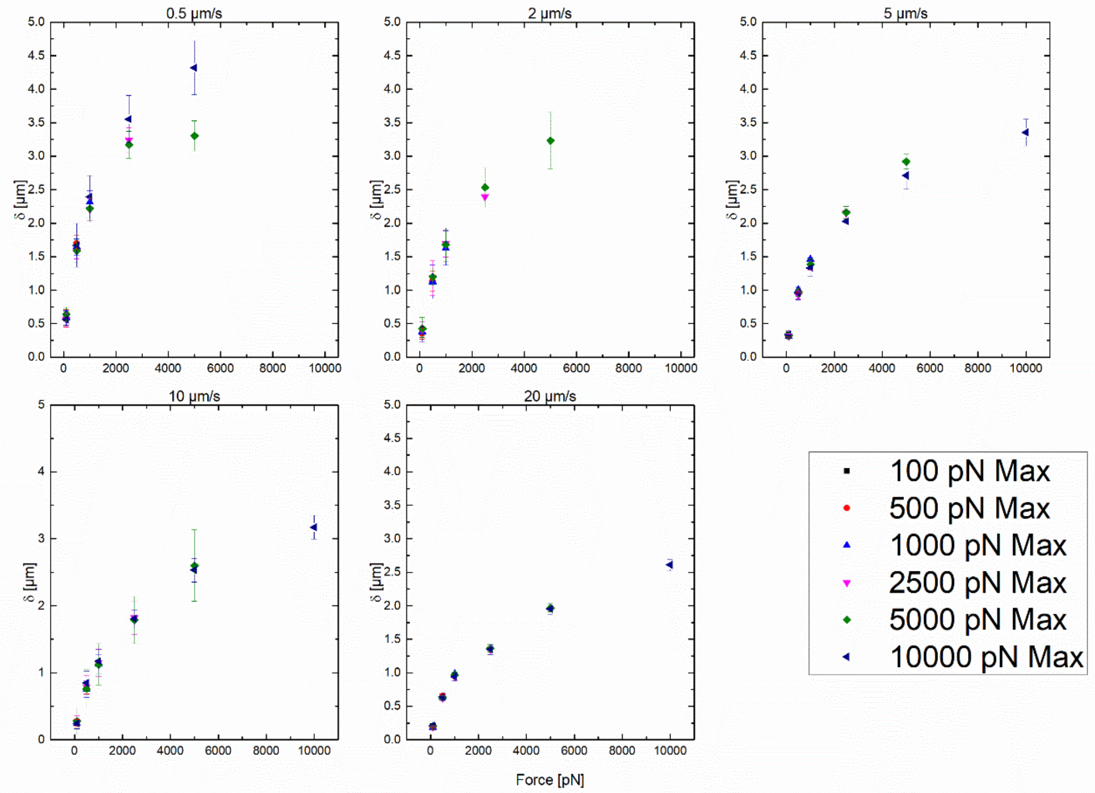

SI.4 – F-d curves for 0.5 µm/s, 10 nN load. The figure shows that at indentations or around 5 nN (with a depth of around 3.5 – 4 µm) a change in slope was visible. This is due to a combined effect of the nucleus (being the stiffest cellular compartment) and the substrate, with the nucleus being squeezed against the substrate.

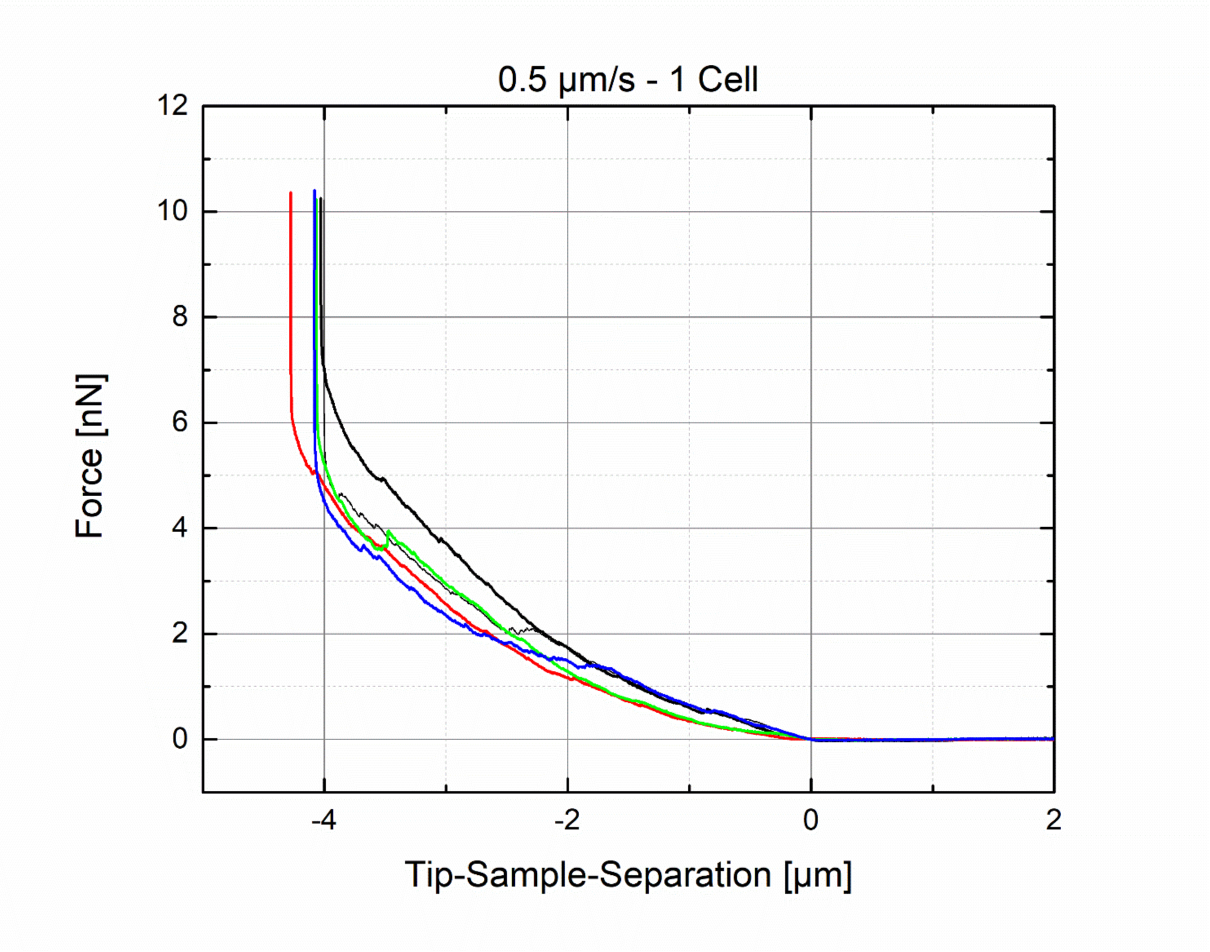

SI.5 – F- δ² curves for 0.5 µm/s, high forces. Representative force-(indentation)² curves for 0.5 µm/s at a force of up to 5 nN. Note the change of the curve of the slope, appearing at around 2.5 nN and a d^2 of 1E-11 m^2 (corresponding to an indentation of around 3 µm). The change is a combination of indenting the nucleus and pressing it against the glass substrate.

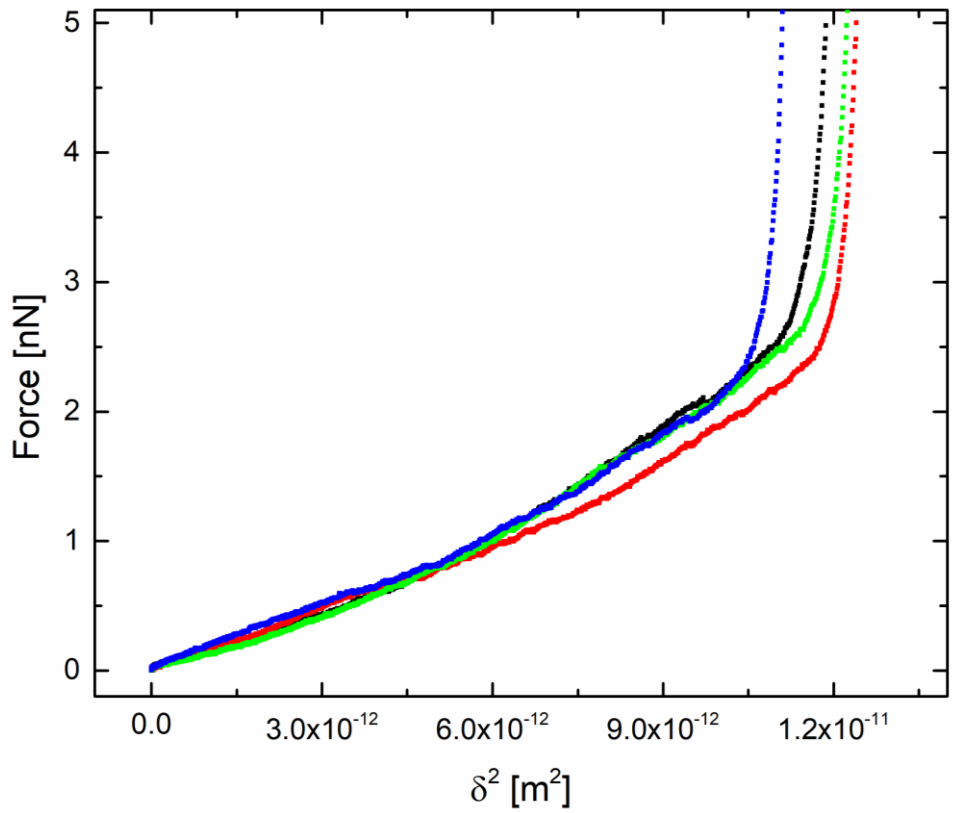

SI.6 – F-δ² curves, closer look at slope changes. The figures show representative curves for low force indentations for 2 µm/s and 5 µm/s, to further evaluate the change of slope. The dashed lines correspond to the two different linear regions. For 2 µm/s, the intercept of both lines lies at a force of around 30 pN, corresponding to an indentation of around 130 nm. For 5 µm/s, it lies at a force of around 25 pN, corresponding to an indentation of around 90 µm. In our opinion, the change of slope results from two effects: i) contact geometry more spherical at very shallow indentations, ii) actin cortex under the membrane of higher stiffness.

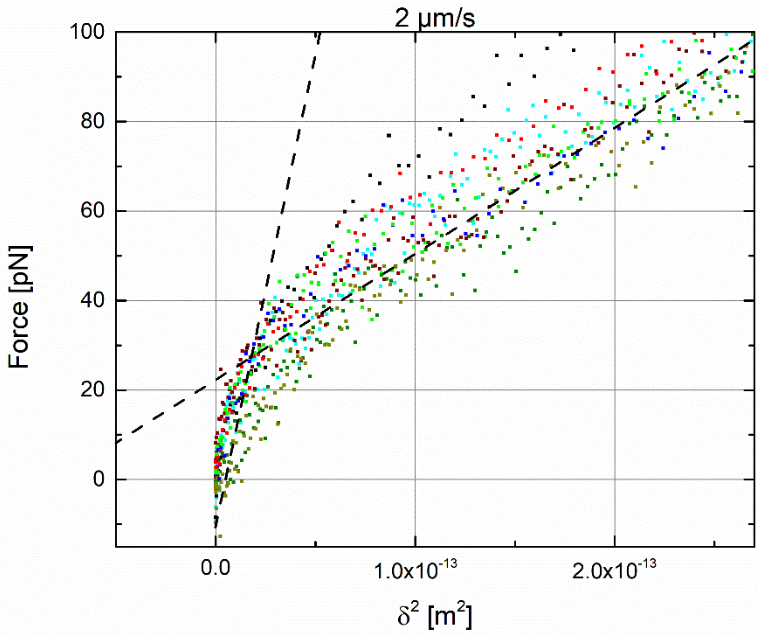

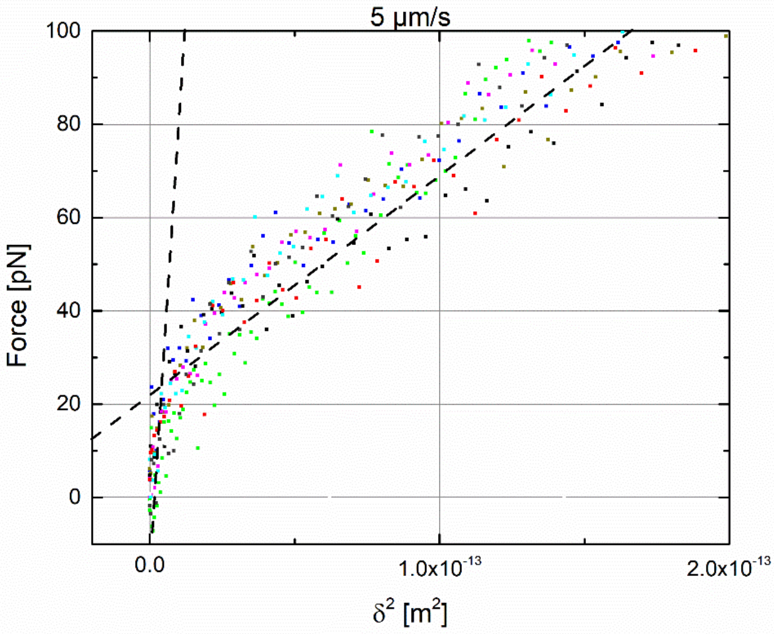

Supplement: Supplementary file 1 — Appendix S1: SUPPLEMENTARY INFORMATION [file JEMT-82-1392-s001.pdf]
